# Hans-Joachim Pflüger: scientist, citizen, cosmopolitan

**DOI:** 10.1007/s00359-022-01550-5

**Published:** 2022-04-25

**Authors:** Carsten Duch, Ansgar Büschges

**Affiliations:** 1 Institute for Developmental Biology and Neurobiology, Johannes Gutenberg-Universität Mainz, Hanns-Dieter-Hüsch-Weg 15, 55128 Mainz, Germany; 2 Institute of Zoology, Biocenter Cologne, University of Cologne, Zülpicher Strasse 47b, 50674 Cologne, Germany

## Abstract

On January 25, 2022, Professor Hans-Joachim Pflüger passed away. Hans-Joachim Pflüger conducted research in the field of neuroethology, with a focus on the development, anatomy, and function of sensorimotor networks underlying insect locomotion. As founding member and one of the presidents of the German Neuroscience Society, Hans-Joachim Pflüger was a driving force behind the development of the Neurosciences in Germany and Europe. This obituary reflects on his curriculum vitae. It shall honor his scientific and professional achievements, and importantly, also his wonderful personality, which makes this loss so sad across the manifold levels of his life and his legacy, the family, the professional and the scientific community.

## Jochen’s career and life in science

Far too early has our colleague, Professor Hans-Joachim ‘*Jochen’* Pflüger, passed away on January 25, 2022, only 72 years old. This loss of our close friend and honorable companion has struck us unexpectedly, because Jochen was highly active, full of infectious energy and exciting future projects. We feel deep sorrow and grief along with his wife, Bärbel, and his family and friends. The numerous ideals, goals, and plans that we have shared with Jochen will remain alive forever.

**Fig. 1:**
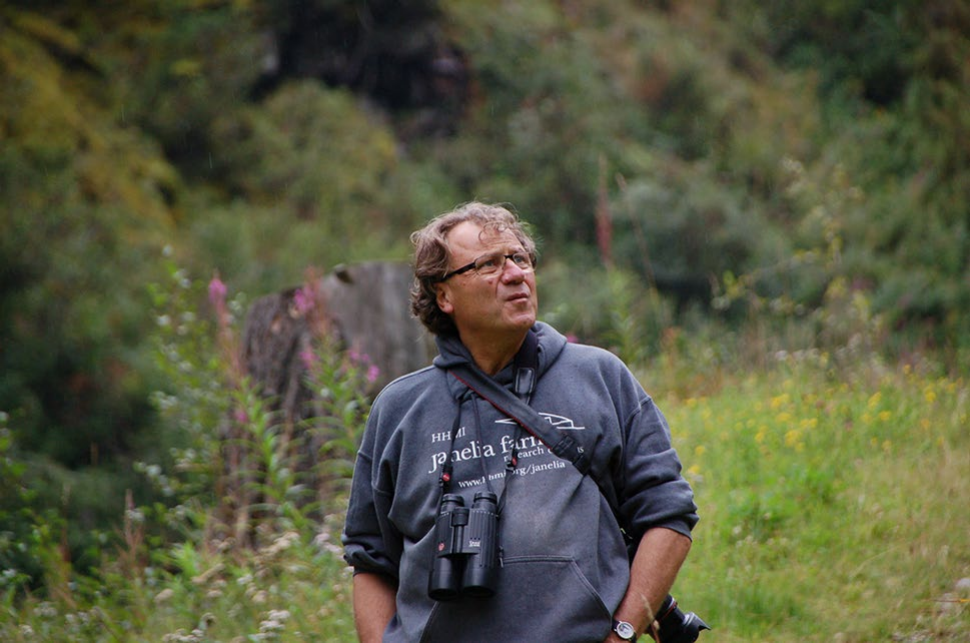
Jochen Pflüger on a hike in the Ahrntal (South Tyrol) in 2014. (Photograph by A. Bueschges)

Jochen (Fig. 1) was born in the post-world war period on March 7, 1949 in Ulm, a medieval town in the southern German state of Baden-Württemberg. There, he completed his primary and high school education and passed his qualification for general university admittance in 1968. Jochen then followed his interests in the natural and life sciences, choosing to study biology and chemistry at the University of Stuttgart, initially with the plan to become a teacher. In 1972, he was selected as “Aufbaustudent” (founding student) with the task of helping to establish a study program for biology at the newly founded University of Kaiserslautern in the state of Rheinland-Pfalz (Rhineland-Palatinate). In 1974, Jochen graduated with the “Staatsexamen” (state examination for teachers) in both chemistry and biology at Kaiserslautern. In the same year, Jochen started secondary school teaching, but in parallel, he engaged in post-graduate studies with Ulrich Bässler, who inspired him to undertake a doctoral thesis on the neural control of limb movements and locomotion, a topic that has fascinated him for decades thereafter. It was in Kaiserslautern where Jochen met his later spouse, Bärbel Kolodziej, a professional costume designer at the municipal theater, at that time operating as a “theater on the road” throughout the Rhineland-Palatinate state.

Jochen’s first scientific study case was the stick insect, *Carausius morosus.* Later he added numerous other animal model species as his attention turned toward a more comparative, evolutionary approach. For his doctoral thesis, Jochen studied the neural control of body “rocking”, a species-specific motor behavior expressed by phasmids for camouflage. Of debate in the field of motor control at that time was the role of neural circuits residing in the central nervous system, so-called central pattern generators (CPGs), capable of generating rhythmic motor activity versus the role that sensory feedback signals play in producing cyclic animal movements and locomotion. In fact, deciphering the relative contributions of these two neural processes remains an issue to be fully resolved for both vertebrate (Grillner and El Manira 2020; Kiehn 2016) and invertebrate motor control (Mantziaris et al. 2020). During his PhD, Jochen demonstrated with skillful experiments that the coordination of motor neuron activity required for stick insect rocking and walking movements can be generated without sensory feedback by a CPG network, but that the centrally generated motor activity is constantly refined by proprioceptive signals that provide feedback information about limb positions and movement velocities (Pflüger 1977). He concluded that both CPG network activity and sensory feedback work hand in hand to control locomotion. This first peer reviewed scientific contribution typified Jochen’s principal attitude: not adhering to pre-existing “dogmas”, but working and reasoning openly to integrate all possible mechanisms contributing to a defined nervous system function.

Jochen completed his PhD in 1976 and then moved for approximately one year to the lab of Malcolm Burrows at the University of Cambridge for his first post doc position studying motor control. Now working with locusts, Jochen started making full use of the ‘identified neuron principle’ in insects, for example, in mapping the axonal projections of mechanosensory neurons onto central motor circuitry. At a time when in vivo genetic labeling, identification, and manipulation of defined types of neurons was not yet feasible, many invertebrate nervous systems offered, with their accessible and relatively small numbers of CNS neurons, the possibility to physiologically and anatomically identify the same neurons from animal to animal. Building on this experimental advantage in insects, Jochen published a series of studies that mapped, both anatomically and functionally, mechanosensory input to identify central motor circuitry (Pflüger et al. 1981, 1986; Pflüger 1984). During this time, he started developing a passion for the direct access to neuronal function by intracellular recordings and through the beauty of neuroanatomy, two aspects of experimental neurobiology that he considered not only to be fundamental methodological tools for scientific advancement, but also in satisfying his quest for innovative artistry. Any student in Jochen’s lab learned quickly under his supervision that neurophysiology and neuroanatomy can be successfully employed according to scientific and esthetic criteria in combination. Jochen followed his interest and fascination in improving his technical and theoretical expertise in studying insect sensorimotor circuits (e.g., Pflüger and Burrows 1987). His relatively brief research period in Cambridge led to subsequent collaborations with his original postdoc supervisor, Malcolm Burrows, over the next four decades, starting with a study on swimming in locusts (Pflüger and Burrows 1978; see also, Pflüger and Burrows (1987) and Burrows and Pflüger (1995)).

In 1977, Jochen returned to Germany, first to the University of Bielefeld (1977–1980) to work in the lab of Peter Görner and subsequently to the University of Konstanz (1980–1987) to the lab of Werner Rathmayer. During this period, Jochen perfected his methodological repertoire for gaining insight into the sensorimotor principles of insect motor control. In 1985 in Konstanz, Jochen passed his habilitation, entitled *Sensorimotor interactions in the locust: Unraveling a mechanoreceptive information path*. In Germany, the habilitation, which is based on an evaluation of both research and teaching achievements, was a mandated PhD follow-up qualification to become eligible for obtaining a university professorship. During this period, Jochen contributed substantially to making the locust a so-called “model organism” in neuroethology, best exemplified by his foundational study on the organization of mechanosensory neuropils in the locust thoracic ganglia that still remains highly relevant for studies on sensorimotor circuit structure and function in insects (Pflüger et al. 1988). While continuing to decipher mechanosensory processing in motor networks in locusts with increasingly elegant and sophisticated methods, in the Rathmayer lab, Jochen also started to analyze the effects of animal toxins and neuromodulatory substances on neuromuscular systems. Today, it is well accepted that in addition to synaptic connections and functional properties of sensorimotor networks, neuromodulation is an essential component for the control of movement by the nervous system (Marder 2012).

Subsequently, in 1987, Jochen accepted the offer of a professorship in *Neurobiology and Functional Neuroanatomy* at the Free University of Berlin, where he conducted his research and taught ever since. Jochen started his own lab focusing on the anatomical and functional characterization of a specific class of aminergic neurons in the locust with central and peripheral projections, so-called dorsal unpaired median (DUM) neurons, named by the location of their unpaired cell bodies at the dorsal midline of the insect CNS (Stevenson et al. 1992; Kononenko et al. 2019). DUM neurons produce and release the biogenic amine, octopamine, the invertebrate analog of norepinephrine in vertebrates (reviewed in Bräunig and Pflüger (2001)). Jochen found that, in addition to the already known functions of biogenic amines, octopamine release from DUM neurons is regulated differentially in different types of DUM cells and is temporally coupled to specific aspects of a motor program (Burrows and Pflüger 1995; Duch et al. 1999). This finding pointed toward more precise roles of aminergic modulation for the generation of motor activity, including locomotion. On the other hand, he showed that task-specific inhibition of specific types of DUM neurons, for example during flight activity of migratory locusts, serves to switch from carbohydrate to lipid metabolism during prolonged flight (Mentel et al. 2003). Overall, Jochen’s studies added important insight into short-term physiological and long-term metabolic adjustments by biogenic amines during insect locomotor behavior.

Jochen’s interest in adaptive changes of neural circuit operation under the influence of neuromodulators led him to team up with colleagues in Berlin who also worked on the functional plasticity of neuronal circuits. Particularly fruitful were his close interactions with his colleague at the Institute of Neurobiology in Berlin, Randolf Menzel, a specialist in the field of insect learning and memory research. This provided the cornerstone for multiple collaborative research consortia that have sparked invertebrate neurobiology research at the Free University of Berlin and beyond. Jochen and Randolf successfully applied for a Research Unit on *Learning, Memory and Neuromodulation in Arthropods* (1989–1995) funded by the German Research Foundation (DFG). Subsequently, Jochen’s research on mechanisms of neuromodulation made an important contribution to the successful installment of the DFG-funded Collaborative Research Center 515 (CRC 515, *Mechanisms of developmental and experience dependent nervous system plasticity*, 1996–2005). This CRC brought together neuroscience groups from multiple Berlin Universities and blue-list Research Institutions. The same inter-institutional initiative held for the DFG-funded Graduate School 837 *Functional Insect Science* in collaboration with the University of Potsdam. Throughout these years, Jochen’s ongoing research contributed to our present understanding of the role of neuromodulation during the development and execution of motor programs and animal behavior in general (Pflüger 1999). In 2010, Jochen finally established a DFG-funded Research Unit on biogenic amine function in insects entitled *Biogenic amines in insects: coordination of physiological processes and behavior*, which he led as the program’s principal coordinator from 2010 to 2016.

Over this period, Jochen transformed his lab at the Königin-Luise-Street in Berlin Dahlem into a well-known and highly attractive location for invertebrate neuroethology colleagues, both nationally and internationally. Scientists from all over Germany and around the globe frequently visited his lab, not only because of the excellent and timely research conducted in his and other labs at the Free University of Berlin, but also because of the open minded, multi-cultural, and warm atmosphere that was present in Jochen’s lab. In fact, this made his lab a virtual hub of German invertebrate neuroethology. His visiting colleagues are too many to cite, but particularly influential to Jochen’s lab were longer visits by Malcom Burrows (University of Cambridge, UK), Richard B “Rick” Levine (University of Arizona, Tucson, US), and Laurence H. “Larry” Field (University of Canterbury, NZ). Viewing Jochen’s list of publications provides a deeper indication to his strength in networking and collaboration. One reflection of Jochen’s international standing was his appointment as adjunct professor at the Arizona Research Laboratories Division of Neurobiology at the University of Arizona, Tucson, AZ since 1991 (Pflüger et al. 1993).

## Jochen—beyond the bench

Jochen was that kind of German university faculty member who was of the strong belief that universities and research institutions need people to actively “run the show” and to assume ownership in building a high-quality professional environment for all other staff associates. In doing so, Jochen contributed to the development of neurobiology and biology at the Free University of Berlin in serving his *alma mater* in various administrative roles, for example, as Managing Director of the Institute, as Dean of the Faculty of Pharmacy, Chemistry and Biology, and as an elected member in the University Senate. At times, these roles were particularly challenging and demanding, for example, during the early 2000s, when the three Berlin universities each had to restructure and reduce their study program portfolios and research positions to accommodate the tremendous financial cut-backs in Berlin at that time.

Jochen’s activities also included supporting high school pupils, students and young researchers in graduate programs, reflected in his reviewing role for *Jugend forscht*, a Germany-wide competition for talented pupils in the areas of science, technology, engineering and mathematics, his role in the DFG-funded Research Training Grant *Signalling Cascades in Living Systems* and his activities in individually mentoring students, for example, recipients of fellowships from the *Studienstiftung des deutschen Volkes (German Academic Scholarship Foundation)*.

Of similar significance was his service to the scientific community in his work for funding agencies, most prominently the DFG, for which he served as member of the neuroscience study section. His broad knowledge in neuroscience that spread into multiple related areas, together with his fair way of judging the work of colleagues, in particular when they were competing with him, made him ideally suited as a reviewer and editorial board member of scientific journals, for example, the *Journal of Comparative Physiology A*, for which Jochen served for more than 2 decades.

Jochen was always highly respected among his colleagues due to his undogmatic view of the different areas of neuroscience research, and his commitment to promoting discovery. This vision made him an excellent participant on boards of national and international scientific associations, such as the *German Neuroscience Society*, the *International Society for Invertebrate Neurobiology*, the *International Society for Neuroethology*, the *Federal European Neuroscience Association* and finally the *International Brain Research Organization (IBRO)*, which organizes the *Global Federation of Neuroscience Societies*. While on all these boards, Jochen served, for example, as treasurer of FENS (2010–12), as member of the Pan-European Research Council (PERC in IBRO) and as treasurer and president of the *German Neuroscience Society* (1992–2003 and 2015–2017, respectively).

We will miss Jochen for many, many other reasons, some of which we would like to highlight here.

**Fig. 2:**
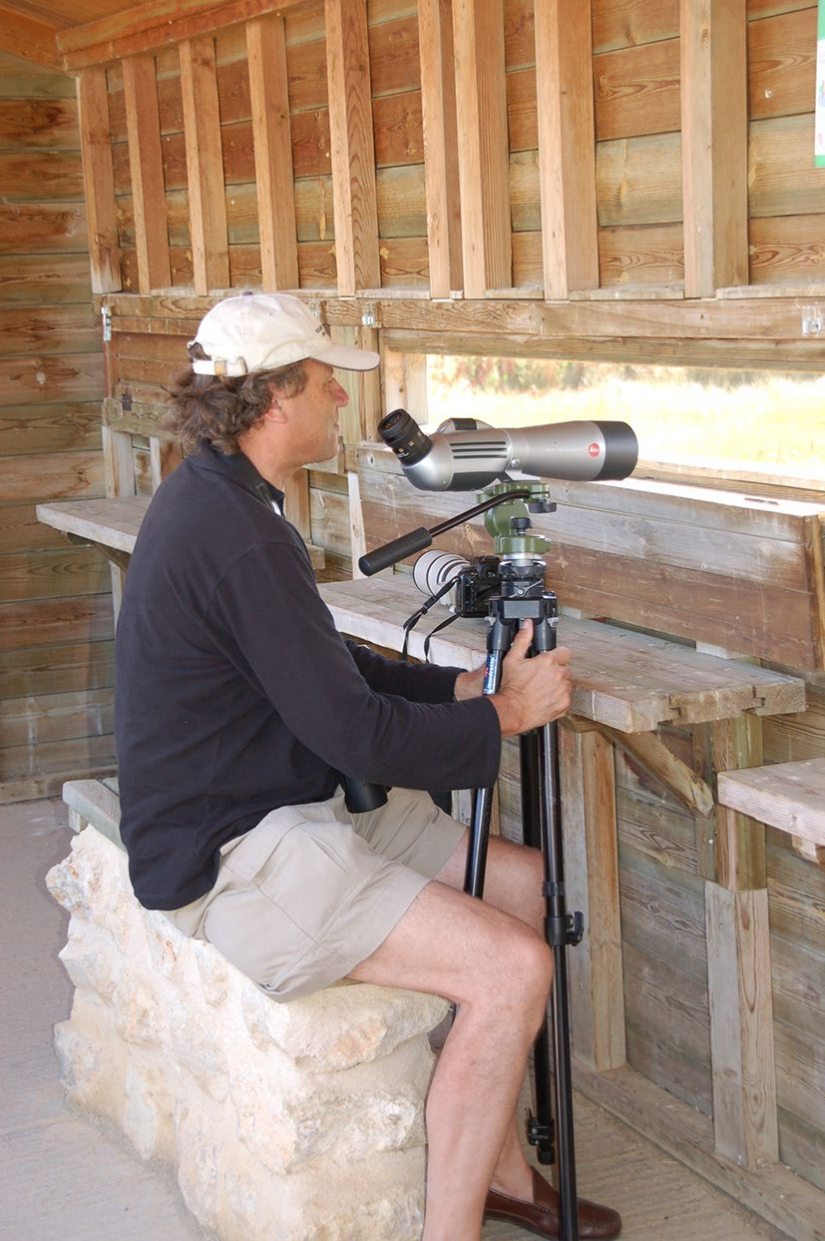
Jochen Pflüger, the ornithologist, in a bird-watching station in the Spanish highlands on the road trip to the 2010 ISN in Salamanca, Spain. (Photograph by A. Bueschges)

Jochen was a true zoologist and neuroethologist, who scrutinized the nervous system of an organism while always retaining and integrating the perspective of the animal’s specific environmental and behavioral setting. For example, when it became known that neural circuits for insect flight and walking differ with respect to their sensitivity to neuromodulators, Jochen chose to compare their systemic actions in winged locust and hawkmoth, as well as in wingless phasmids. Jochen’s broad biological knowledge was known to those who had the chance to participate and observe him on one of his excursions for bird-watching (Fig. 2). These outings were not only regularly offered in the surrounds of Berlin to students, colleagues, and friends, but he also planned and embarked on them with companion scientists throughout the globe, ranging from visits to the ornithological station at the Baltic Curonian Spit to excursions after international neurobiology meetings. For example, in traveling by car to the congress of the International Society for Neuroethology in Salamanca, Spain in 2010, Jochen had planned the trip in such a way that it essentially turned out to be a major bird-watching excursion of some 1800 km!

Working at the Free University of Berlin served the cultural interest of Jochen perfectly. When living in Berlin, Jochen and Bärbel always were highly reliable and expert informants on which exhibition to visit, which concert to enjoy and which theater play to attend. Many of us had the immense pleasure of diving into the cultural life of Berlin with Jochen and Bärbel when a visit to Berlin was on the agenda. In the same way, Jochen’s own collaborative visits around the globe were always accompanied by his exploration of the local cultural life and customs.

Jochen cared in a marvelous, impressive way about the history of science and society. Two examples illustrate this aspect of his personality: In 2016, Jochen was awarded the first *Ernst Bresslau Professorship* at the Institute of Zoology at the University of Cologne. Ernst Bresslau, the Institute’s first Director in 1925, was expelled by the Nazis in 1933 based on their “Gesetz zur Wiederherstellung des Berufsbeamtentums” (law to reinstate professional officialdom) and his quarter Jewish heritage, even though he was a highly decorated military doctor during World War I. Prof. Bresslau had to leave Germany permanently with his family and took up the founder position for the Institute of Zoology at Sao Paulo in Brazil. Jochen not only honored the visiting professorship by his stay at the Institute and his close collaboration with Institute colleagues, but he also looked closely into the work and life of Ernst Bresslau (Pflüger 2017). In a fascinating way, Jochen used all possible sources of information, such as archives, media, and institutions, to collect information about the Institute’s founder and his fate during the Nazi period. Jochen even visited Ernst Bresslau’s relatives in Sao Paulo to inspect the original hand-written excursion books from the latter’s various field trips almost 100 years ago. These efforts led to an unexpected view on the life of the founder of the Institute of Zoology at the University of Cologne. One of Jochen’s last contributions to our understanding of the history of zoology was a collaboration with Julia Heideklang and Helmut Kettenmann to translate the dissertation of Hermann Helmholtz from Latin into English and German (Heideklang et al. 2021). This early work of Helmholtz, entitled *De fabrica systematis nervosi evertebratorum* attracted Jochen’s passion for fine neuroanatomy, since already in 1842, it provided major insights into invertebrate neuroanatomy that still hold today.

We will always keep Jochen in our memories and hearts as an outstanding investigative scientist, an all-round biologist, a companion, close friend, and a true citizen of the world.

## References

[CR1] Bräunig P, Pflüger H-J (2001). The unpaired median neurons of Insects. Adv Insect Physiol.

[CR2] Burrows M, Pflüger H-J (1995). The action of locust neuromodulatory neurons is coupled to specific motor patterns. J Neurophysiol.

[CR3] Duch C, Mentel T, Pflüger H-J (1999). Distribution and activation of different types of octopaminergic DUM neurons in the locust. J Comp Neurol.

[CR4] Grillner S, El Manira A (2020). Current principles of motor control, with special reference to vertebrate locomotion. Physiol Rev.

[CR5] Heideklang J, Pflüger HJ, Kettenmann H (2021). Commented thesis by Herman Helmholz.

[CR6] Kiehn O (2016). Decoding the organization of spinal circuits that control locomotion. Nat Rev Neurosci.

[CR7] Kononenko NL, Hartfil S, Willer J, Ferch J, Wolfenberg H, Pflüger H-J (2019). A population of descending tyraminergic/octopaminergic projection neurons of the insect deutocerebrum. J Comp Neurol.

[CR8] Mantziaris C, Bockemühl T, Büschges A (2020). Central pattern generating networks in insect locomotion. Dev Neurobiol.

[CR9] Marder E (2012). Neuromodulation of neuronal circuits: back to the future. Neuron.

[CR10] Mentel T, Duch C, Stypa H, Müller U, Wegener G, Pflüger H-J (2003). Central modulatory neurons control fuel selection in flight muscle of migratory locust. J Neurosci.

[CR11] Pflüger H-J (1977). The control of the rocking movements of the phasmid *Carausius morosus*. Br J Comp Physiol A.

[CR12] Pflüger H-J (1984). The large fourth abdominal intersegmental interneuron: a new type of wind-sensitive ventral cord interneuron in locusts. J Comp Neurol.

[CR13] Pflüger H-J (1999). Neuromodulation during motor development and behavior. Curr Opin Neurobiol.

[CR14] Pflüger H-J (2017). Professor Ernst Bresslau, founder of the Zoology Departments at the Universities of Cologne and Sao Paulo: lessons to learn from his life history. Zoology.

[CR15] Pflüger H-J, Burrows M (1978). Locusts use the same basic motor pattern in swimming as in jumping and kicking. J Exp Biol.

[CR16] Pflüger H-J, Burrows M (1987). A strand receptor with a central cell body synapses upon spiking local interneurones in the locust. J Comp Physiol A.

[CR17] Pflüger H, Bräunig P, Hustert R (1981). Distribution and specific central projections of mechanoreceptors in the thorax and proximal leg joints of locusts. Cell Tissue Res.

[CR18] Pflüger H-J, Elson R, Binkle U, Schneider H (1986). The central nervous organization of the motor neurons to a steering muscle in locusts. J Exp Biol.

[CR19] Pflüger H-J, Bräunig P, Hustert R (1988). The organization of mechanosensory neuropiles in locust thoracic ganglia. Philos Trans R Soc Lond B.

[CR20] Pflüger H-J, Witten J, Levine RB (1993). Fate of abdominal ventral unpaired median cells during metamorphosis of the hawkmoth, *Manduca sexta*. J Comp Neurol.

[CR21] Stevenson PA, Pflüger H-J, Eckert M, Rapus J (1992). Octopamine immunoreactive cell populations in the locust thoracic-abdominal nervous system. J Comp Neurol.

