# Editorial Expression of Concern: Hans-Joachim Pflüger: scientist, citizen, cosmopolitan

**DOI:** 10.1007/s00359-022-01589-4

**Published:** 2022-11-11

**Authors:** Carsten Duch, Ansgar Büschges

**Affiliations:** 1 Institute for Developmental Biology and Neurobiology, Johannes Gutenberg-Universität Mainz, Hanns‑Dieter‑Hüsch‑Weg 15, 55128 Mainz, Germany; 2 Institute of Zoology, Biocenter Cologne, University of Cologne, Zülpicher Strasse 47b, 50674 Cologne, Germany

On January 25, 2022, Hans-Joachim Pflüger, a former Associate Editor of the *Journal of Comparative Physiology A*, passed away. To commemorate his life and work, the Editor-in-Chief, Günther K. H. Zupanc, invited Carsten Duch and Ansgar Büschges to write an obituary for the *Journal*. The submitted manuscript underwent peer and editorial review before a revised version was published online on April 25, 2022 (Duch and Büschges 2022a). However, based on this manuscript version, an essentially identical obituary had appeared 2 weeks earlier, on April 11, 2022, in *Neuroforum* (Duch and Büschges 2022b), a journal edited by the *Neurowissenschaftliche Gesellschaft e.V.* and published by Walter de Gruyter GmbH. Neither the Editor-in-Chief nor the publisher of the *Journal of Comparative Physiology A*, Springer Nature, had obtained prior knowledge of, or given consent for, the intended dual publication.

The authors acknowledged receipt of this Editorial Expression of Concern but did not comment on its content.
